# Multiparameter MRI Radiomics Model Predicts Preoperative Peritoneal Carcinomatosis in Ovarian Cancer

**DOI:** 10.3389/fonc.2021.765652

**Published:** 2021-10-21

**Authors:** Xiao Yu Yu, Jialiang Ren, Yushan Jia, Hui Wu, Guangming Niu, Aishi Liu, Yang Gao, Fene Hao, Lizhi Xie

**Affiliations:** ^1^ Affiliated Hospital, Inner Mongolia Medical University, Hohhot, China; ^2^ Department of Pharmaceuticals Diagnosis, GE Healthcare (China), Shanghai, China; ^3^ Department of Radiology, Inner Mongolia International Hospital, Hohhot, China

**Keywords:** ovarian cancer, peritoneal carcinomatosis, radiomics, magnetic resonance imaging, predictions

## Abstract

**Objectives:**

To evaluate the predictive value of radiomics features based on multiparameter magnetic resonance imaging (MP-MRI) for peritoneal carcinomatosis (PC) in patients with ovarian cancer (OC).

**Methods:**

A total of 86 patients with epithelial OC were included in this retrospective study. All patients underwent FS-T2WI, DWI, and DCE-MRI scans, followed by total hysterectomy plus omentectomy. Quantitative imaging features were extracted from preoperative FS-T2WI, DWI, and DCE-MRI images, and feature screening was performed using a minimum redundancy maximum correlation (mRMR) and least absolute shrinkage selection operator (LASSO) methods. Four radiomics models were constructed based on three MRI sequences. Then, combined with radiomics characteristics and clinicopathological risk factors, a multi-factor Logistic regression method was used to construct a radiomics nomogram, and the performance of the radiomics nomogram was evaluated by receiver operating characteristic curve (ROC) curve, calibration curve, and decision curve analysis.

**Results:**

The radiomics model from the MP-MRI combined sequence showed a higher area under the curve (AUC) than the model from FS-T2WI, DWI, and DCE-MRI alone (0.846 *vs*. 0.762, 0.830, 0.807, respectively). The radiomics nomogram (AUC=0.902) constructed by combining radiomics characteristics and clinicopathological risk factors showed a better diagnostic effect than the clinical model (AUC=0.858) and the radiomics model (AUC=0.846). The decision curve analysis shows that the radiomics nomogram has good clinical application value, and the calibration curve also proves that it has good stability.

**Conclusion:**

Radiomics nomogram based on MP-MRI combined sequence showed good predictive accuracy for PC in patients with OC. This tool can be used to identify peritoneal carcinomatosis in OC patients before surgery.

## Introduction

Ovarian cancer (OC) is the fifth most common cancer in women and the most common gynecological tumor. Epithelial ovarian cancer (EOC) is the most common OC subtype accounting for 90% of all OC. It is characterized by extensive and rapid intra-abdominal carcinomatosis and has a poor prognosis and high mortality. The 5-year survival rate of EOC is only 30% (1–4). If the patient can detect PC at an early stage, it will be able to buy sufficient treatment time for the patient and effectively control the patient’s condition from further deterioration. Preoperative detection of peritoneal carcinomatosis (PC) is essential to avoid unnecessary resection and choose the best treatment method for patients with EOC.

National Comprehensive Cancer Network guidelines recommend that all peritoneal surfaces suspected of carcinomatosis should be selectively removed. Many ovarian cancers do not have ascites when they have peritoneal carcinomatosis. Because there are many peritoneal folds, smaller peritoneal metastatic nodules can be easily misdiagnosed, which affects the treatment and prognosis of patients. The diagnosis of peritoneal implants mainly relies on open exploratory surgery and laparoscopy; nonetheless, the existing results are hardly consistent. In addition, laparoscopic surgery, which is invasive and expensive, carries certain risks, such as intraoperative tumor capsule rupture, incision carcinomatosis (5, 6). Therefore, there is an urgent need for an accurate non-invasive technique to assess the PC’s condition.

As an alternative method, computed tomography (CT) is usually used for preoperative examination. However, CT has limited sensitivity and may easily overlook carcinomatosis below 1 cm (7). Radiomics, an emerging and promising research field based on quantitative imaging technology, can provide decision support for oncology by extracting high-throughput quantitative radiological features from medical images (8). This low-cost and non-invasive technique has been successfully used for tumor diagnosis, staging, treatment monitoring, and treatment plan formulation (9–12). This method has been successfully applied to preoperatively predict peritoneal carcinomatosis of gastric cancer (13, 14) and may be potentially used for ovarian cancer. So far, no personalized prediction model has been developed for peritoneal carcinomatosis of ovarian cancer. This study evaluated the value of multi-parameter MRI radiomics in predicting preoperative peritoneal carcinomatosis in patients with ovarian cancer. We established a combined clinical-radiomics model to help improve decision-making and guide individualized treatment.

## Materials and Methods

### Patient Information

This retrospective study was approved by the ethical review committee of our hospital, and informed consent was obtained from patients.

**Figure 1:**
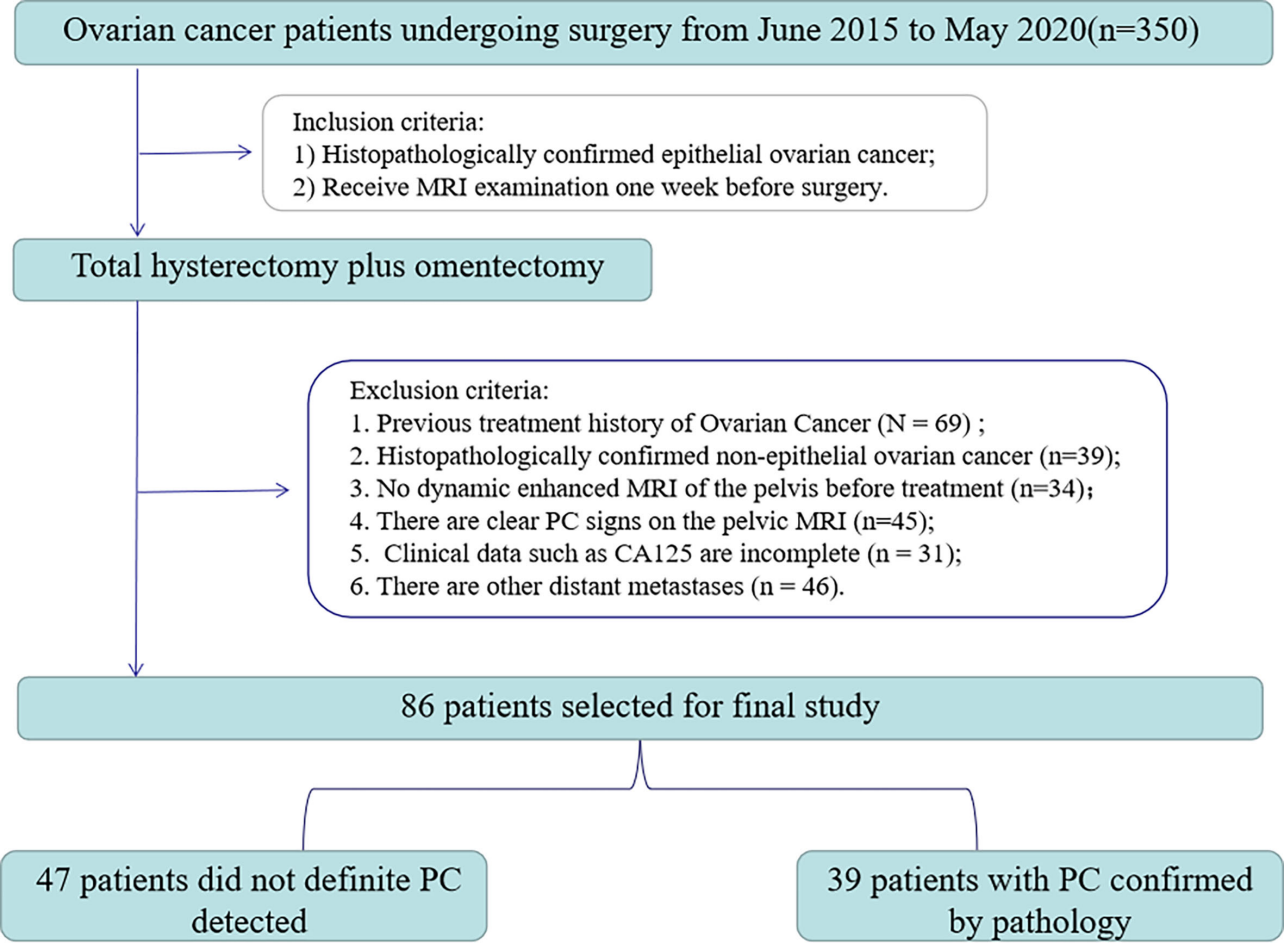
Flowchart of patient selection.

From June 2015 to May 2020, 350 consecutive EOC patients were retrieved retrospectively in our hospital’s image archiving and communication system (PACS, GE). The inclusion/exclusion criteria and patient recruitment process are shown in **Figure 1**. Inclusion criteria: 1) Histopathologically confirmed epithelial ovarian cancer; 2) Receive MRI examination one week before surgery. Exclusion criteria were: 1) past treatment history of ovarian cancer (n=69); 2) the histopathology was non-epithelial OC (n=39); 3) no dynamic enhanced MRI of the pelvis before treatment (n=34); 4) presence of clear PC signs on pelvic MRI (n=45); 5) the clinical data of CA125 were incomplete (n=31); 6) there are other distant carcinomatosis (n=46). Finally, 86 patients (age 33-82, median age 54) were enrolled in the study.

All patients underwent full hysterectomy with double appendages and increased omentectomy. The existence of PC was unanimously determined by pathologists and gynecologists according to AJCC (American Joint Committee on Cancer) guidelines. Finally, 39 out of 47 patients were detected with peritoneal carcinomatosis. The clinicopathological characteristics, including age, preoperative CA125 level, abdominal symptoms, menopausal history, genetic history, and type, were obtained from patients’ medical records.

Two radiologists with 3 and 15 years of experience in female pelvic MRI imaging, who were blind to the pathological results but knew whether the patient was diagnosed with EOC, reviewed the MRI images and recorded the following: (1) unilateral or bilateral ovarian tumors; (2) tumor size (the volume of the largest layer); (3) T2 signal (low and high signals were divided by the signal strength near the myometrium); (4) enhancement degree. The radiological characteristics were selected according to the criteria of Guo HL et al. (15). Bilateral lesions were determined using the same pathological type according to the pathological surgical results; the largest tumor is finally selected for analysis. The ADC value was obtained according to the method of Thomassin et al. (16). The clinical and tumor characteristics of the patients are summarized in **Tables 1**, **2**.

**Table 1:** Single-factor analysis of clinicopathological characteristics of 86 EOC patients.

Features	Without PC	With PC	p value
Number of patients	47	39	
Age (mean ± SD, years)	51.7 ± 8.8	56.8 ± 10.6	0.017
CA125 (median ± IQR, μ/ml)	213.1 (75.0-397.4)	1237. (608.7-2247.9)	<0.001
Genetic history (%)			0.950
Yes	7 (14.9%)	6 (15.4%)	
No	40 (85.1%)	33 (84.6%)	
Menopause(%)			0.381
Yes	27 (57.4%)	26 (66.7%)	
No	20 (42.6%)	13 (33.3%)	
Abdominal symptoms (%)			0.988
Yes	29 (61.7%)	24 (61.5%)	
No	18 (38.3%)	15 (38.5%)	
Type (%)			0.087
Type I	19 (40.4%)	9 (23.1%)	
Type II	28 (59.6%)	30 (76.9%)	

SD, standard deviation; IOR, interquartile range; ADC, apparent diffusion coefficient; PC, peritoneal metastasis.

**Table 2:** Single-factor analysis of MR imaging characteristics of 86 EOC patients.

Features	Without PC	With PC	p value
Size (median ± IQR, mm3)	884.7 (239.9-1123.8)	596.4 (120.0- 844.2)	0.093
ADC (Average ± SD, mm2/s)	0.001 ± 0.001	0.001 ± 0.000	0.088
Location (%)			0.123
Unilateral	34 (72.3%)	22 (56.4%)	
Bilateral	13 (27.7%)	17 (43.6%)	
T2 homogeneity (%)			0.203
Low	14 (29.8%)	7 (17.9%)	
High	33 (70.2%)	32 (82.1%)	
T1 enhancement (%)			0.057
Mild	20 (42.6%)	9 (23.1%)	
Obvious	27 (57.4%)	30 (76.9%)	

SD, standard deviation; IOR, interquartile range; ADC, apparent diffusion coefficient; PC, peritoneal metastasis.

### Imaging Acquisition and Preprocessing

All MRI examinations were performed on a 3.0T system (Discovery MR750, GE Healthcare), using an 8-channel phased body coil. Before scanning, the patient with moderately filled bladder was placed in a supine position. Patients were also asked to fast 4-6 hours before the examination, and intestinal preparation (lactulose and magnesium sulfate) was used to reduce bowel movements. Scans were then performed along the pubic bone to the iliac spine. MR scanning parameters on the 3.0-T scanner DCE imaging of the pelvis was performed after administration of 0.1 mmol/kg of body weight of gadolinium chelate (Gadovist; Bayer). Images were acquired at postcontrast enhancement 120 seconds in the axial plane. This protocol obtained axial FS-T2WI, DWI, and DCE-MRI images. Detailed information about the acquisition parameters is shown in **Table S1**.

Before image segmentation, preprocessing was required. First, according to the research of Qian et al. (17), the fs-T2WI and DWI sequence images of each patient were selected for registration to the DCE-MRI (late arterial stage only) image. Then, the planar resolution of each mode was uniformly resampled to 1x1x1mm. Finally, the method of Cohen (18) was applied to normalize the image contrast of each mode to correct the factors that may affect the intensity unevenness. All the processing was performed on the 3D Slicer (version 4.10.2, funded by the National Institutes of Health) software.

### MRI Radiomics Feature Extraction and Selection

Ovarian cancer lesions were performed by two radiologists with 3 years (A) and 15 years (B) experience in abdominal imaging respectively on each layer of DWI (b=1000s/mm2) to perform 3D manual manipulation of the primary tumor along the edge of the lesion segmentation. The region of interest (ROI) covered the entire tumor. The FS-T2WI and DCE-MRI were compared to avoid the cystic, necrotic, or hemorrhage area of the tumor (see **Appendix 1**).

**Figure 2:**
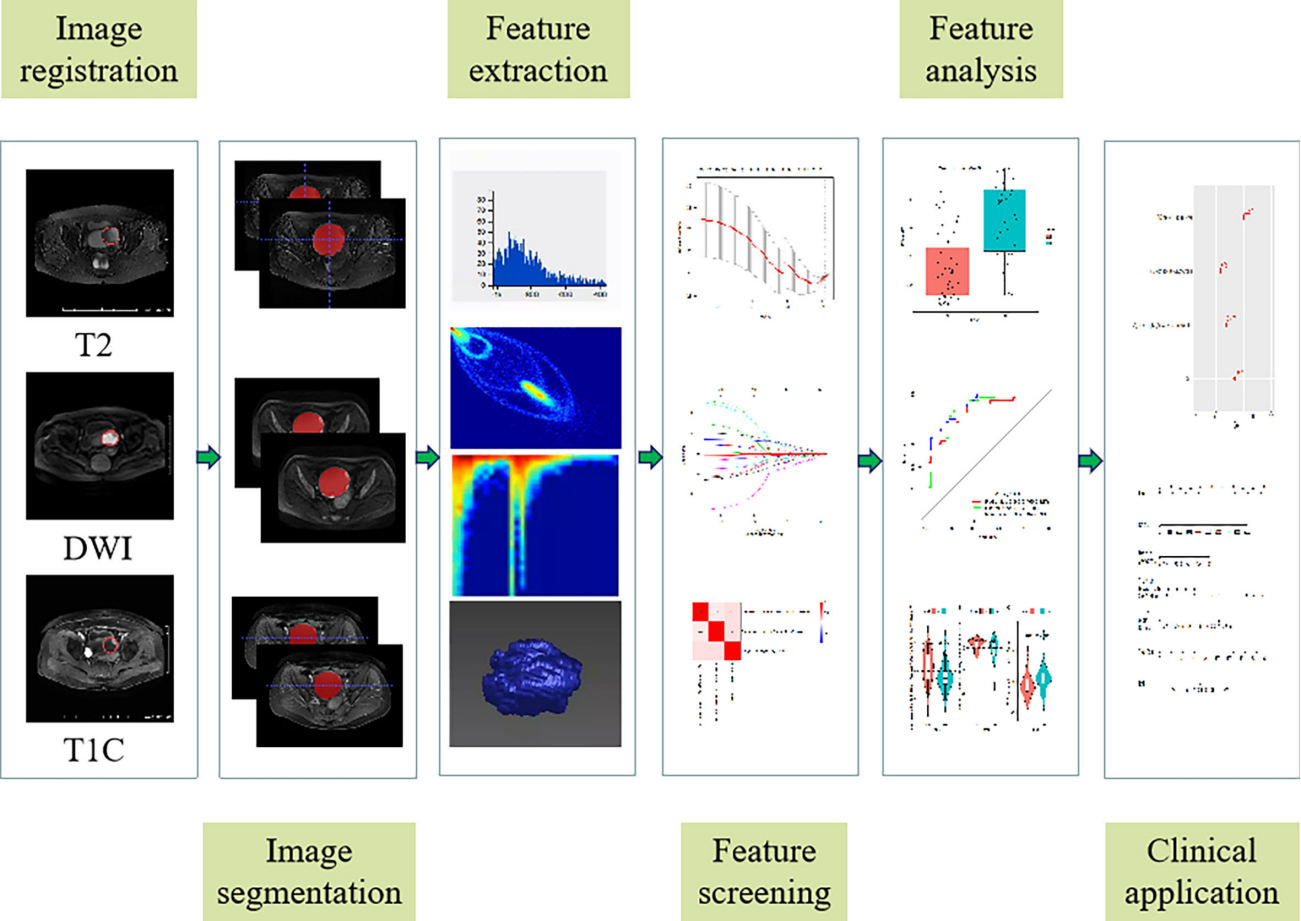
Radiomics signature workflow.

PyRadiomics was used (19) to extract the radiologic signatures. Wavelet (8 filtering parameters) and Laplace of Gaussian (LoG, 2 filtering parameters) transformations were applied on the original image, respectively. Then, 1037 features were extracted from 11 different image types, including (1) gray histogram features; (2) morphological features; (3) gray level co-occurrence matrix (GLCM) features; (4) gray level run length matrix (GLRLM) functions; (5) grayscale area matrix (GLSZM) features. After that, all eigenvalues were normalized using Z-Score transformation. In order to ensure the reproducibility of the model results and reduce the over-fitting or selection bias in the radiomics model, the intra- and inter-group correlation coefficient (ICC) was used to evaluate the characteristics of retention stability and high repeatability, and the ICC threshold was set to 0.75. Then, the minimum redundancy maximum correlation (mRMR) was used to sort the remaining features, and each sequence retained the best top 20 features (20–22). Next, the least absolute shrinkage selection operator (LASSO) (23) method was used to screen the radiological features used to evaluate PC status. Finally, multi-factor stepwise logistic regression was used, and the Akaike Information Criterion (AIC) was used as the stopping condition to determine the best combination of radiological characteristics and clinical data (23). The workflow is shown in **Figure 2**.

### Establishment of Radiomics and Clinical Models

The radiomic characteristics screened by the above method were incorporated into the multivariate Logistic regression analysis to establish a radiomics model. All the above steps were performed on the radiomics model extracted from FS-T2WI, DWI, and DCE-MRI separately and the combined model. In addition, for comparison, a Logistic regression analysis model containing clinical data was also established. Finally, the radiomics features were combined with clinical data to construct a hybrid model. In order to provide visualization and a personalized tool for predicting the probability of ovarian cancer peritoneal carcinomatosis, we have drawn a nomogram. The calibration curve was used to evaluate the calibration of the nomogram, and the Hosmer-Lemeshow test was performed. Decision curve analysis was used to calculate the net income of different models under different threshold probabilities.

### Statistical Analysis

All statistical analyses were performed using R (Version 3.6.3, Statistical Computing Basis). Independent sample t-test or Mann-Whitney U test were used to examine the differences in measurement data uses, and the chi-square test or Fisher’s exact test to evaluate the count data differences. The receiver operating characteristic curve (ROC) of the model was drawn, and the area under the curve (AUC) and 95% confidence interval (CI) were calculated to quantify the discriminant ability of the model. Delong test was used to compare the AUC among different models. The diagnostic sensitivity, specificity, accuracy, positive likelihood ratio, and negative likelihood ratio were also examined. Calibration curves were used to evaluate the predictive performance of each model. Decision curve analysis (DCA) was used to evaluate the net benefits of each model under different threshold probabilities and to evaluate the clinical applicability of each model. A two-sided p-value less than 0.05 was considered to be statistically significant.

## Results

### Clinical and Tumor Characteristics of Patients

The clinicopathological and radiological characteristics of the patients are shown in **Tables 1** and **2**. There were significant differences in age and preoperative CA125 levels between ovarian cancer (OC) with PC and OC without PC (p=<0.001-0.017). However, no differences were found for abdominal symptoms, menopausal history, genetic history, type, lesion location, tumor size, T2 signal, and ADC between the two groups.

### Evaluation of Radiomics

According to the standard of ICC>0.75, FS-T2WI, DWI, and DCE-MRI sequences retained 508, 557, and 508 radiomic features, respectively. Minimum redundancy maximum correlation and Lasso regression were then performed on the selected omics features to adjust penalty parameters through 10 cross-validation and select non-zero coefficient features related to PC status. Finally, 2 FS-T2WI features, 1 DWI feature, and three DCE-MRI features were used to build a model (**Appendix Figure 2**). Consequently, after removing features by multivariate Logistic regression, 3 features were retained, and a combined multi-sequence model was established (**Appendix Figure 3**).

**Figure 3:**
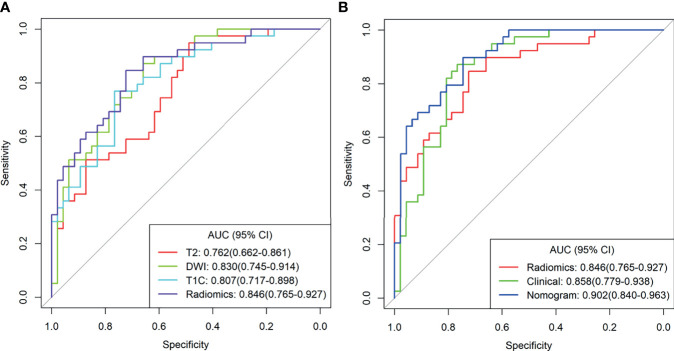
**(A)** T2WI radiation model, DWI radiation model, T1C Radiation Model, and combined radiation model. **(B)** Clinical Model, combined radiological model, and Nomogram Receiver Operating Characteristic curve.

The combined model from multiple sequences showed better distinguishing ability than the single model (when using a single sequence). The ROC curves of the four models are shown in **Figure 3A**. The AUC values of Fs-T2WI, DWI, CE-T1WI, and the combined model were 0.762 (0.662-0.861), 0.830 (0.745-0.914), 0.807 (0.717-0.898), and 0.846(0.765-0.927), respectively. The performance of the models is shown in **Table S2**. The DCA curves and calibration curves of the four models are shown in **Appendix Figure 4**.

### Model Comparison and Nomogram Performance

**Figure 4:**
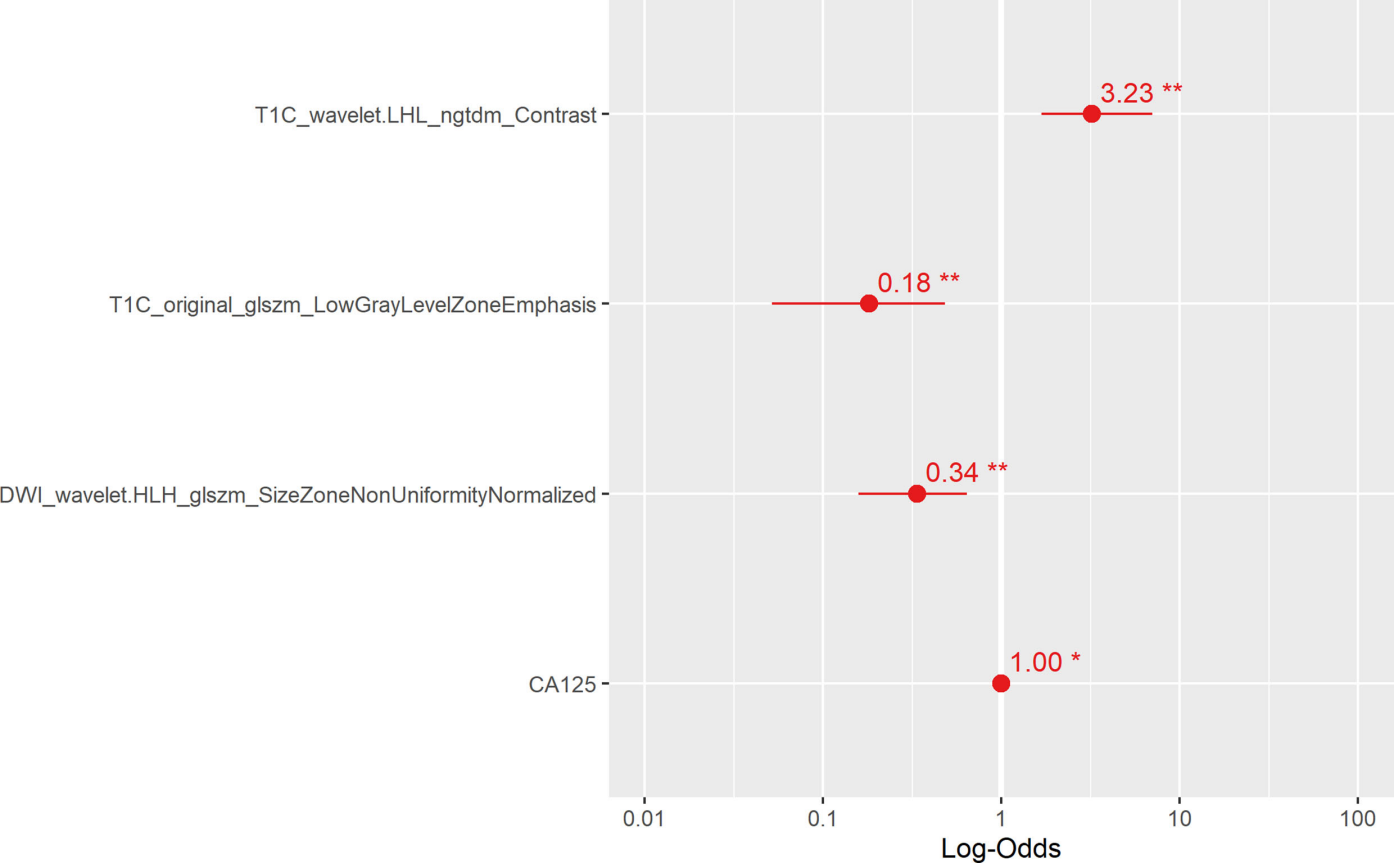
The main radiological features extracted in this study and the results of multiple logistic regression of preoperative CA125. The horizontal line is the 95% confidence interval of the study, and the small dot in the center of the horizontal line is the point of the OR value.

**Figure 5:**
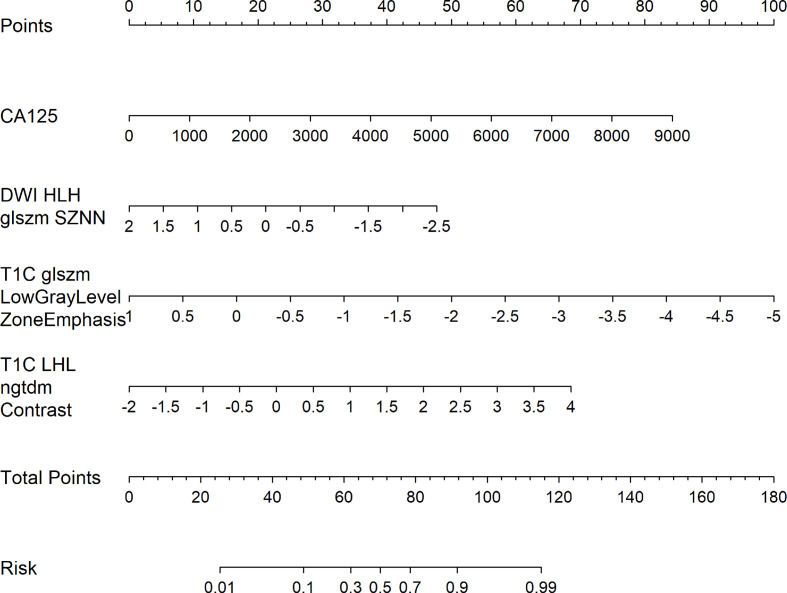
Radiology nomogram. The radiology nomogram prediction model predicts the probability of PC in patients with epithelial ovarian cancer. The model is developed in a training group with radiomic characteristics and one clinical feature. How to use: (1) locate the patient’s CA125 and then draw a straight line on the top dot axis to obtain a score related to CA125; (2) the patient’s radiologic score is found on the characteristic axis of Radiology, and a line is drawn vertically up along the “point” axis. The process is repeated for each variable. (3) Sum up the sum of the four major risk factors. (4) Find the final sum on the Total Point axis and draw a straight line down to assess PC’s risk in patients with epithelial ovarian cancer.

Multivariate analysis of clinical data and radiomic characteristics showed that preoperative CA125 level, DWI_HLH_glszm_SizeZoneNonUniformityNormalized, T1C_glszm_LowGrayLevelZoneEmphasis, T1C_LHL_ngtdm_Contrast were significant predictors (**Figure 4**). As a result, they were fused into a nomogram (**Figure 5**). The AUC of the radiology nomogram was higher than that of the clinical model and the radiomic model (0.902, 95%CI: 0.846-0.858), indicating that the radiology nomogram can effectively distinguish the presence or absence of peritoneal carcinomatosis. The predictive performance of the clinical model was not significantly different from that of the omics model (AUC=0.858 *vs*. AUC=0.846). **Figure 3B** summarizes the diagnostic performance and ROC analysis results of these three models.

**Figure 6:**
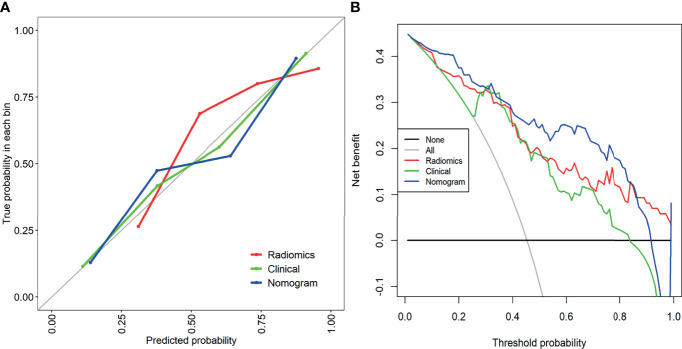
**(A)** The calibration curve of the clinical model, combined radiology model, and nomogram. It is a curve with the model predicted PC probability as the X-axis and the actual PC probability as the Y-axis. The degree of coincidence between the calibration curve depicted and the 45-degree straight line reflects the predictive performance of each model. **(B)** Decision curve analysis of the clinical model, combined radiomics model, and mixed model. The Y-axis represents net income. The blue line represents the radiographic nomogram. Red lines represent radiomics models. The green line represents the model that contains only clinical features. The gray line represents the assumption that all patients have LN metastasis. The thin black line indicates the hypothesis that no patients have PC metastasis.

The calibration curve of the nomogram showed a good agreement between the predicted value and the observed value. The Hosmer-Lemeshow test was not significant (p>0.05), indicating a good degree of fit (**Figure 6A**). Decision curves were used to compare the benefits of nomograms, radiomics models, and clinical models, and we found that when the threshold probability of DCA curves was 37%-85%, nomograms had better predictive performance than clinical models and omics models (**Figure 6B**).

## Discussion

In this study, a nomogram radiomics model for preoperative prediction of peritoneal carcinomatosis of EOC was proposed based on clinical data and radiomics features reflecting primary tumors’ characteristics. Our research shows that the multi-sequence combination model is better than the single-sequence model. The constructed nomogram provides an easy-to-use, non-invasive and individualized tool for PC diagnosis and provides decision support for clinicians.

Some studies have explored the value of MRI in the evaluation of PC in epithelial ovarian cancer. At present, all published studies, including recent studies, have been conducted with comparable or fewer patient sample sizes, mainly focusing on routine imaging (15, 24, 25). Our study extracted more than 3000 features from MP-MRI images and evaluated the MR imaging features of patients with epithelial ovarian cancer in FS-T2WI, DWI, DCE-MRI, and the combination of the three. When FS-T2WI, DWI, and DCE-MRI were combined, the diagnostic efficiency of the presence or absence of peritoneal carcinomatosis was the highest, which is consistent with previous reports in the literature (26, 27). The DWI of our study is very close to the AUC of radiomics. This is because in the absence of ascites, some small lesions are usually better seen on the DWI image than on the standard T1 and T2 weighted images. To the best of our knowledge, this is the first study that combines anatomical, diffusion, and perfusion MRI and uses radiomics analysis based on the primary tumor to predict PC. Therefore, the MP-MRI combined model reveals more detailed tumor information and can more accurately predict ovarian cancer’s PC status.

In our study, preoperative CA125 level was considered to be an independent predictor of PC carcinomatosis. Age has a certain potential for predicting PC, but it is not as effective as CA125. Therefore, a clinical model based on CA125 was established. Our data showed that the risk of PC with high levels of CA125 was significantly higher, which is consistent with the results reported in the previous literature (28, 29). At the same time, in order to facilitate clinical use, we have developed a nomogram containing preoperative CA125 levels and radiomic characteristics, with an AUC value of 0.902, for predicting PC. The DCA curve showed a satisfactory net income. The calibration curve showed that the stability was slightly inferior to the clinical model. This is because the clinical model was established based on a single variable, and CA125 was a continuous variable with a small fluctuation range. The nomogram integrates clinical data and radiomics characteristics and contains multi-dimensional quantitative and detailed information, so the results obtained are more objective and accurate.

Peritoneal carcinomatosis are common in the stomach, gallbladder, pancreas, lungs, intestines, uterus, and ovaries (30–32). Most of the patients with early-stage ovarian cancer PC have no specific symptoms; these patients are usually diagnosed when in an advanced stage. It is one of the main causes of ovarian cancer morbidity and mortality (32). So far, many studies have evaluated the PC status of patients with ovarian cancer (15, 24, 25). CT is a common tool used to detect PC, but its missed diagnosis rate is high. Although ^18^F-FDG PET/CT has been shown to achieve good results in evaluating PC status, it is not widely used because of the high cost. MRI has a high resolution for soft tissues and can clearly show anatomical relationships.

Studies have recently shown that radiomics can predict peritoneal carcinomatosis in cancer patients (13, 14). Dong et al. (13) developed a personalized nomogram to identify the occult peritoneal carcinomatosis of advanced gastric cancer, achieving good results, which is similar to our findings, with few differences: first, we used MP-MRI to extract features, which can reflect tumor information more comprehensively and in more detail; second, we established a single sequence model and a combined model. Through comparison, we found that the combined model had better performance in predicting PC. In addition, the nomogram we established also showed good clinical practicability and ability to provide a diagnostic basis for predicting the PC status of ovarian cancer before clinical surgery.

This study has a few limitations. First, it is a retrospective study with small sample size. In the future, more patients are needed to provide more reliable evidence for clinical applications. Secondly, the research model’s establishment and verification is a single-center, and further data from multiple centers are needed for external validation. In addition, we only performed radiomics analysis on late-arterial phase of contrast-enhanced MRI. Perhaps venous MRI images may provide more useful radiomics information, which needs to be discussed further. Finally, only primary tumors were selected in our study, and the radiohistological features of the peritoneum were not routinely used. MRI texture analysis of peritoneum needs to be further studied to explore its value.

In summary, we established nomograms based on preoperative CA125 and radiographic characteristics from primary tumors, which can be used to predict peritoneal carcinomatosis in EOC patients preoperatively. This effective and easy-to-use new approach provides a non-invasive and reliable tool for EOC patients to develop individualized treatment plans.

## Data Availability Statement

The original contributions presented in the study are included in the article/**Supplementary Material**. Further inquiries can be directed to the corresponding authors.

## Ethics Statement

The studies involving human participants were reviewed and approved by Biomedical Medical Research Ethics Committee of Inner Mongolia Medical University. The patients/participants provided their written informed consent to participate in this study.

## Author Contributions

XY and JR substantial contributions to the conception or design of the work; or the acquisition, analysis or interpretation of data for the work. HW and GN drafting the work or revising it critically for important intellectual content. YJ, FH, and LX provide approval for publication of the content. YG and AL agree to be accountable for all aspects of the work in ensuring that questions related to the accuracy or integrity of any part of the work are appropriately investigated and resolved. All authors contributed to the article and approved the submitted version.

## Funding

This article was funded by the Inner Mongolia Autonomous Region Fund of Natural Science (2021MS08026).

## Conflict of Interest

Author JR was employed by GE Healthcare.

The remaining authors declare that the research was conducted in the absence of any commercial or financial relationships that could be construed as a potential conflict of interest.

## Publisher’s Note

All claims expressed in this article are solely those of the authors and do not necessarily represent those of their affiliated organizations, or those of the publisher, the editors and the reviewers. Any product that may be evaluated in this article, or claim that may be made by its manufacturer, is not guaranteed or endorsed by the publisher.

## Abbreviations

OC, ovarian cancer; EOC, epithelial ovarian cancer; PC, peritoneal carcinomatosis; AJCC, American Joint Committee on Cancer; MP-MRI, multiparameter magnetic resonance imaging; ROI, region of interest; ICC, inter-group correlation coefficient; AUC, area under the curve; DCA, decision curve analysis.
