# Supplementary material for: Multiparameter MRI Radiomics Model Predicts Preoperative Peritoneal Carcinomatosis in Ovarian Cancer
Source: Front Oncol. 2021 Oct 21;11:765652. doi: 10.3389/fonc.2021.765652 (PMC8591658; doi:10.3389/fonc.2021.765652)
Supplement: Supplementary file 1 [file DataSheet_1.doc]

***Table S1 MR imaging parameters***

|  | TR/TE  (ms) | Field of  view(cm) | Number   of slices | Slice   thickness | Slice   gap(mm) | NEX | Bandwidth   (HZ/px) | Matrix | b Value  (sec/mm2) |
| --- | --- | --- | --- | --- | --- | --- | --- | --- | --- |
| Fs-T2WI | 3429/68 | 32 | 20 | 5 | 0.2 | 2 | 125 | 512*256 | N |
| DWI | 2400/20 | 32 | 20 | 5 | 0.2 | 2 | 125 | 128*256 | 0, 1000 |
| T1C | 2.8/1.3 | 36 | 20 | 5 | 0.2 | 2 | 125 | 320*192 | N |

*Note: The MR scan parameters for DCE imaging of the pelvis on the 3.0-T scanner were performed after the injection of 0.1 mmol/kg body weight of gadolinium chelate (Gadovist; Bayer). The image was acquired at postcontrast enhancement 120 seconds in the axial plane.*

**Table S2. Predictive performance of FS-T2WI, DWI, DCE-MRI, radiomics combined model and clinical model, nomogram**

| Feature number | Method | AUC(95%CI) | ACC(95%CI) | SEN(95% CI) | SPE(95% CI) | PPV(95% CI) | NPV(95%CI) |
| --- | --- | --- | --- | --- | --- | --- | --- |
| 2 | T2 | 0.762(0.662-0.861) | 0.698(0.589-0.792) | 0.974(0.717-1.000) | 0.468(0.127-0.617) | 0.603(0.528-0.609) | 0.957(0.857-0.967) |
| 3 | DWI | 0.830(0.745-0.914) | 0.756(0.651-0.842) | 0.872(0.640-0.949) | 0.660(0.382-0.787) | 0.680(0.610-0.698) | 0.861(0.782-0.881) |
| 3 | T1C | 0.807(0.717-0.898) | 0.767(0.664-0.852) | 0.769(0.410-0.872) | 0.766(0.532-0.872) | 0.732(0.592-0.756) | 0.800(0.735-0.820) |
| 3 | Radiomics | 0.846(0.765-0.927) | 0.779(0.677-0.861) | 0.846(0.589-0.949) | 0.723(0.468-0.830) | 0.717(0.639-0.740) | 0.850(0.786-0.867) |
| 2 | Clinical | 0.858(0.779-0.938) | 0.814(0.716-0.890) | 0.872(0.563-0.974) | 0.766(0.553-0.872) | 0.756(0.666-0.776) | 0.878(0.839-0.891) |
| 5 | Nomogram | 0.944(0.901-0.988) | 0.884(0.797-0.943) | 0.795(0.614-0.923) | 0.957(0.744-1.000) | 0.939(0.923-0.947) | 0.849(0.814-0.855) |

*Note: AUC: area under curve; SEN: sensitivity; SPE: specificity; ACC: accuracy; PPV: positive predictive value; NPV: negative predictive value*

*
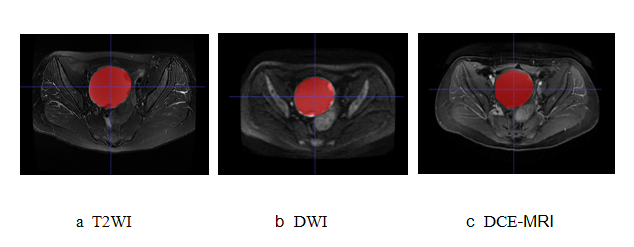
*

***Fig. S1 VOI drawing method****. Two radiologists performed 3D manual segmentation of the primary tumor along the edge of the lesion at each layer of DWI (b=1000s/mm2), ROI covered the whole tumor, and finally, the volume of interest (VOI) of the whole tumor was obtained.*

***
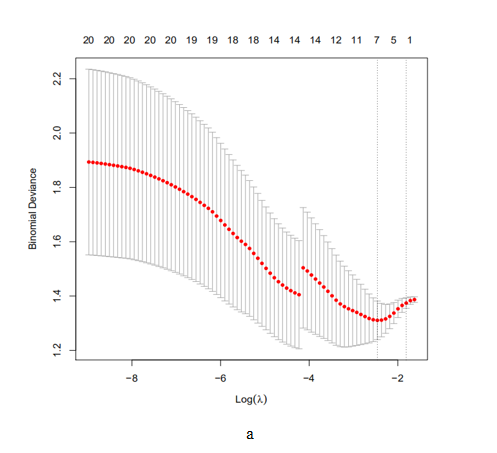
***

***
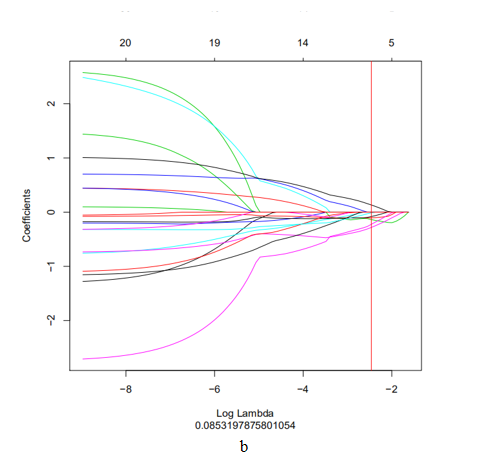
***

***Figure S2. Use minimum absolute shrinkage and selection operator (lasso) regression method for radiomic feature selection.*** ***(a)*** *Determine the optimal penalty coefficient λ(λ) in the lasso model with 10 times of cross-validation and the minimum criterion. In this study, the λ value was set to 0.08531979****. (b)*** *Draw a dashed vertical line at the selected λ value, resulting in 5 non-zero coefficient features.*

*
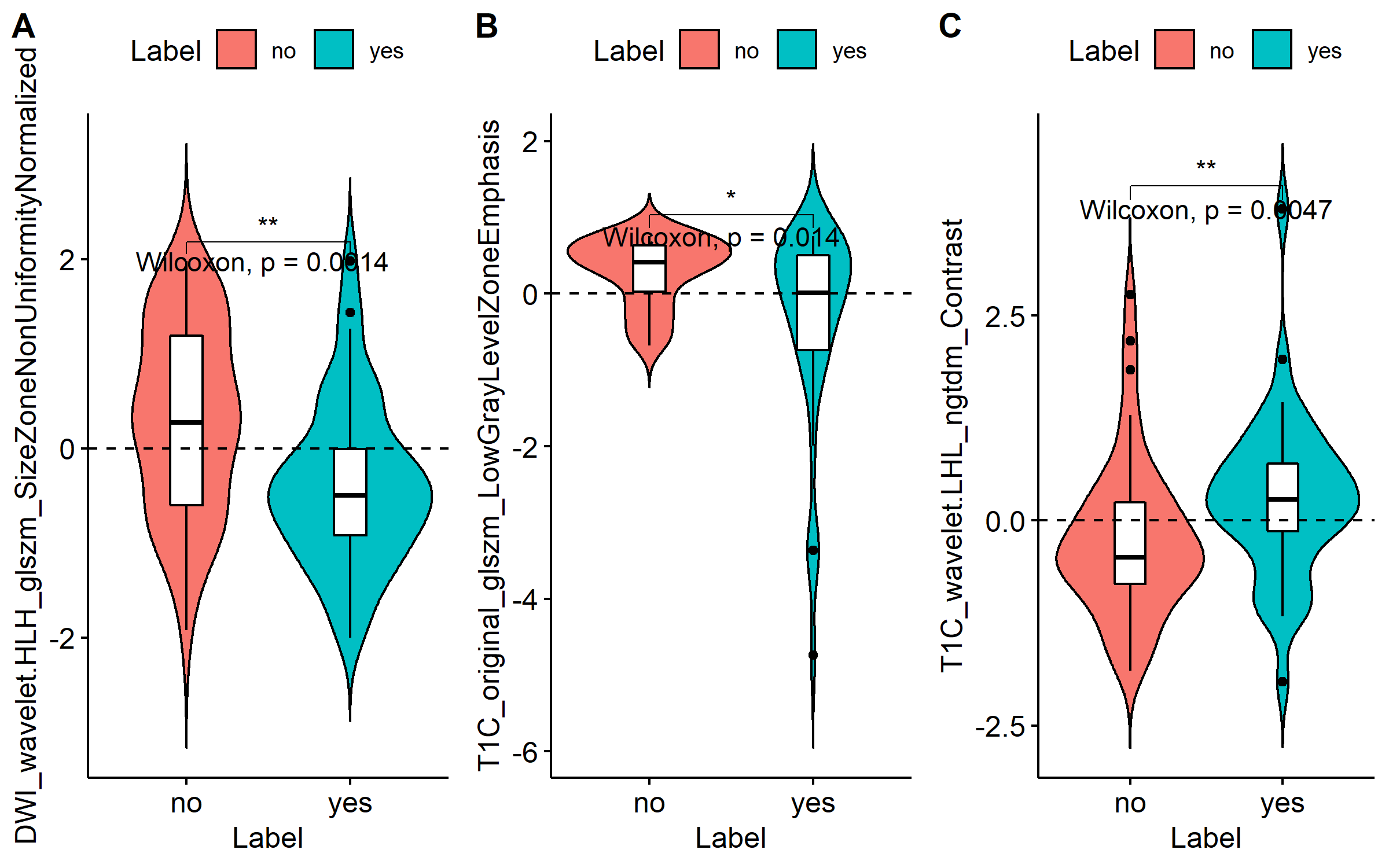
*

***Fig. S3 Multivariate Logistic regression analysis to obtain 3 radioomics characteristics****. The middle black line is the median, the middle white thick bar represents the interquartile range, and the thin black line extending from the thick white bar represents 95 % Confidence interval. Lable represents the label, red is negative for peritoneal metastasis, and green is positive for peritoneal metastasis*

*
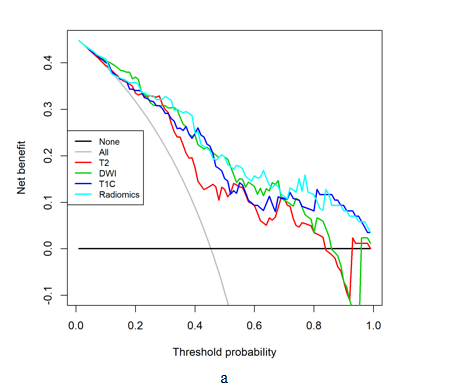
*

*
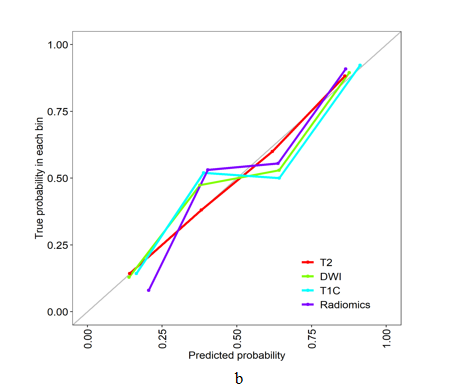
*

***Fig. S4  DCA curve and a calibration curve of four models. (a)*** *DCA Curves of Four Models.* ***(b)*** *Scale curves of four models.*
